# Adaptation to acute pulmonary hypertension in pigs

**DOI:** 10.14814/phy2.13605

**Published:** 2018-03-07

**Authors:** Mare Mechelinck, Marc Hein, Sven Bellen, Rolf Rossaint, Anna B. Roehl

**Affiliations:** ^1^ Department of Anesthesiology Medical Faculty RWTH Aachen University Aachen Germany

**Keywords:** Coronary circulation, myocardial contraction, physiological adaptation, pulmonary hypertension, right, swine, ventricular dysfunction

## Abstract

The extent of right ventricular compensation compared to the left ventricle is restricted and varies among individuals, which makes it difficult to define. While establishing a model of acute pulmonary hypertension in pigs we observed two different kinds of compensation in our animals. Looking deeper into the hemodynamic data we tried to delineate why some animals could compensate and others could not. Pulmonary hypertension (mean pressure 45 mmHg) was induced gradually by infusion of a stable thromboxane A_2_ analogue U46619 in a porcine model (*n* = 22). Hemodynamic data (pressure‐volume loops, strain‐analysis of echocardiographic data and coronary flow measurements) were evaluated retrospectively for the short‐term right ventricular compensatory mechanisms and limits (Roehl et al. [2012] Acta Anaesthesiol. Scand., 56:449–58) 10 animals showed stable arterial blood pressures, whereas 12 pigs exhibited a significant drop of 16.4 ± 9.9 mmHg. Cardiac output and heart rate were comparable in both groups. In contrast, right ventricular contractility and coronary flow only rose in the stable group. The unchanging values in the decrease group correlated with an increasing ST‐segment depression and a loss of ventricular synchronism and resulted in a larger septum bulging to the right ventricle. Simultaneously, a reduced left‐ventricular end‐diastolic volume and a missing improvement in contractility in the posterior septal and inferior free wall of the left ventricle have been observed. Our findings suggest that right ventricular compensation during acute pulmonary hypertension is strongly dependent on the individual capability to increase coronary flow. The cause for inter‐individual variability could be the dimension and reactivity of the coronary system.

## Introduction

Pulmonary hypertension (PHT) in a perioperative and intensive care setting is accompanied by a significantly increased risk for postoperative complications and a high mortality rate (Reich et al. 1999; Fischer et al. 2003; Lai et al. 2007; Forrest 2009; Kaw et al. 2011; Vonk‐Noordegraaf et al. 2013) Therefore, there is a need for effective prevention, detection, and treatment of PHT, and consequently, the need for better pathophysiological knowledge becomes clear. Previous research showed that sufficient adaptation of the right ventricle (RV) to acute PHT through an increase in contractility is a crucial process that influences patient survival (Bristow et al. 1998; Vonk‐Noordegraaf et al. 2013). The exact factors this adaptation depends on are not clear yet. However, it is known that the RV due to its thinner wall has a reduced contractile reserve compared to the left ventricle (LV) (Zwissler 2000).

Pulmonary hypertension is defined as mean pulmonary artery pressure (mPAP) greater than 25 mmHg at rest (Opitz et al. 2016). This change leads to an increasing RV afterload. The resulting pressure overload and dilatation of the RV that usually starts at end‐diastolic pressure values of 40 mmHg (Ama et al. 2006) triggers a rise in RV contractility (de Vroomen et al. 2000; Mebazaa et al. 2004; Rex et al. 2007). This so‐called homeometric autoregulation, or Anrep effect, leads to an imbalance between the increased RV wall tension with an associated elevated oxygen consumption and a reduced oxygen supply due to the decreased perfusion pressure. Depending on the extent of the discrepancy, this can result in myocardial ischemia and a subsequent reduction in contractility. The vicious circle can end up in right heart failure and subsequently lead to cardiac decompensation.

To evaluate the exact cardiac movement analysis of strain rate imaging is an established noninvasive method. The septal peak systolic strain, the absolute value of the septal strain and the strain rate are markers of contractility and have been shown to be reduced in patients with PHT (Pirat et al. 2006).

A crucial aspect of PHT development is its progress over time: long‐term elevation of pulmonary pressure with consequent vascular remodeling has clearly higher critical values than an acute increase in pulmonary pressure. For instance, the long‐term increase can be due to sepsis, valvular defects, acute respiratory distress syndrome or chronic obstructive pulmonary disease, whereas a short‐term elevation is more frequently caused by different forms of embolization, administration of protamine, hypoventilation or hypoxia (Smulders 2000; Forrest 2009). With our experimental setup, we focused on the acute type of PHT.

In addition, coronary perfusion could be crucial because it forms the basis for any changes in contractility. Due to lower systolic pressure and less wall stress, coronary perfusion occurs during the whole cardiac cycle in the RV (Crottogini et al. 1991; Zong et al. 2005).

In cases of rising RV pressure, the overload induces a nitric oxide (NO) release that leads to coronary vasodilatation and maintains coronary blood flow (CBF) (Zong et al. 2005). This mechanism only works up to a certain point at which the wall tension gets too high, and consequently, the perfusion pressure and the contractility decrease. The critical perfusion pressure that marks the lower limit of this autoregulatory range depends on the characteristics of the left versus the right coronary branches. It is lowest in the RV free wall (30 mmHg) and highest in the LV third of the septal wall (45 mmHg) (Guth et al. 1991). Above these limits, an increase in the tissue pressure of 10 mmHg decreases CBF by 30 percent (Gibbons Kroeker et al. 2006), and severe PHT can even cause temporary retrograde CBF (Lowensohn et al. 1976).

Because human hearts consist of two circuits, studying RV function without considering LV influence is not reasonable. Ventricular interaction in PHT is manifold, and yet it is poorly understood. Systolic and diastolic influences have been previously described (Goldstein 2002). In addition, both a “serial” interaction through changes in flow quantity due to the Frank‐Starling mechanism and a “parallel” or “pericardial” interaction are discussed (Ama et al. 2006; Chua et al. 2013). In response to PHT, both ventricles show a reduction in their active relaxation and their compliance (Ama et al. 2006). However, the importance of the Frank‐Starling mechanism for the RV itself is debatable because the right heart seems to be more sensitive to changes in afterload than in preload (Ama et al. 2006).

Considering ventricular interaction, the septal wall plays a crucial role and ensures right heart function in patients suffering from PHT (Goldstein 2002; Bleeker et al. 2006). Physiologically, LV septal contraction performs one‐third of the RV stroke work (Goldstein 2002). During PHT, a paradoxical shift of the septal wall has been observed that suggests that septal movement for the RV function is more important than previously thought (Goldstein 2002; Bleeker et al. 2006; Chua et al. 2013). The established explanation for this observation is that an increase in RV afterload during PHT causes alterations in RV geometry, followed by changes in electrical conductivity. This process leads to prolonged RV contraction (Vonk‐Noordegraaf et al. 2005; Marcus et al. 2008; Handoko et al. 2009; Hardziyenka et al. 2009). The consequent asynchronous action of the ventricles causes a reversed transseptal pressure gradient, and thus, a bulging of the septal wall into the LV occurs during early LV diastole (Vonk‐Noordegraaf et al. 2005; Ama et al. 2006; Marcus et al. 2008; Handoko et al. 2009; Hardziyenka et al. 2009). Therefore, shortening of the septal muscle fibers in systole generates a paradoxical septal movement to the RV (instead of to the LV) along with an increased RV ejection (Goldstein 2002). This mechanism is crucial for the remaining RV function.

In our animal experiments, we gradually induced PHT in pigs (Roehl et al. 2012) and observed two types of reactions. Some pigs were able to adapt to PHT, and others were not. Animals that could not adapt showed signs of right heart failure. Therefore, we decided to analyze this inter‐individual variability further.

The aim of this study was to better understand the onset of acute PHT and to determine the factors that can possibly predict or influence different reactions to PHT.

## Methods

### Animals

All experiments in this study were approved by the local animal care committee and the governmental animal care office (No. 50.203.2‐AC38,4/05 Landesamt für Natur‐, Umwelt und Verbraucherschutz Nordrhein‐Westfalen, Germany). The protocols were designed according to the *Guide for the care and use of laboratory animals* (Institute of Laboratory Animal Resources CoLS, National Research Council, 1996). Roehl et al. (2012) published findings concerning these animal experiments earlier, so further information relating to the protocol can be found there.

For this study, data from 22 female breeding pigs (German Landrace) with a mean weight of 30.2 ± 1.45 kg (mean ± standard deviation) were evaluated retrospectively. In these pigs, PHT was induced in stages using a continuous infusion of thromboxane A_2_ analogue U46619 though the central venous catheter. For this study, 13 pigs had to be excluded due to missing data concerning the exact PHT interstages.

### Anesthesia

After an acclimation period of 5 days and overnight fasting, the animals received an intramuscular premedication of azaperone (4 mg/kg) and intravenous propofol (3 mg/kg) for general anesthesia. Oral intubation followed, and anesthesia was maintained with a continuous infusion of thiopental (15 mg/kg/h). No muscle relaxants were given. Volume‐controlled mechanical ventilation was performed with 21% oxygen with a tidal volume of 10 ml/kg, a positive end‐expiratory pressure of 7 mbar and a breathing rate of 20–24 per minute (Physioflex^®^; Draeger, Luebeck, Germany). Ringer's solution was infused (10 ml/kg/h), and active heating was used as needed to maintain normothermia (38.5°C). Cardiac rhythm, oxygen saturation and invasive femoral arterial pressure were continuously monitored during experimental procedures (S/5; Datex‐Ohmeda, Helsinki, Finland).

### Instrumentation

After preparing the right cervical vessels, a conductance catheter was placed through the common carotid artery and the aortic valve towards the apex of the LV (SPR‐570‐7; Millar Instruments, Houston, TX, USA). A pressure sensor was placed at a distance of approximately 5 cm from the apex.

To be able to provoke a short‐term LV preload reduction, a balloon occlusion catheter was placed in the inferior vena cava via the right femoral vein.

After median sternotomy and opening of the pericardium, ultrasound transit‐time flow probes were placed proximally around the right coronary artery (RCA) (MA 2.5 PSB; Transonic, Maastricht, Netherland) and the main pulmonary artery (MA 20 PAX; Transonic). An RV conductance catheter was inserted via a small puncture site at the RV apex, and a pressure sensor was introduced via an RV wound in the RV outflow tract with the tip of the sensor located 3–4 cm distally from the pulmonary valve (CA‐61000‐PL; CDLeycom, Zoetermeer, Netherlands).

After successful positioning of the catheters, a break of approximately 2 h was taken so that the hemodynamic parameters could recover from manipulation, and stable baseline parameters could be established.

### Protocol

To induce PHT within 45 min in 5 mmHg stages between a mPAP of 20 mmHg and 45 mmHg, stable thromboxane A_2_ analogue U46619 (1 *μ*g/ml; Cayman Chemical, Ann Arbour, MI, USA) was continuously administered as an infusion with increasing dosage (0.2 to 0.8 *μ*g/kg/min).

### Monitoring, measurement, and calculations

Mean arterial pressure (mAP), mean pulmonary artery pressure (mPAP), and heart rate (HR) were recorded continuously. Cardiac output (CO) was calculated from pulmonary arterial flow measurements.

Conductance catheters were used to collect continuous, real‐time recordings of the intraventricular pressures and volumes (such as the Ved). A calibration of the volume measurement was carried out by injecting 5 ml of 10% intravenous saline solution at each measurement point. The exact calculation has been described in previous studies (Baan et al. 1984; Steendijk and Baan 2000; Danton et al. 2002). Data for creating pressure‐volume loops were acquired in apnea. These loops were used to calculate the arterial elastance and TAU.

Coronary blood flow was determined during intervals of 10–20 sec. Arterial and coronary venous oxygen saturations were additionally measured, so that myocardial oxygen extraction and MvO_2_ could be calculated. Furthermore, ST depressions were recorded over time for subsequent analysis.

Echocardiography was performed in 17 animals at low (20–25 mmHg) and high (40–45 mmHg) levels of mPAP (Vivid I; General Electric Co., Horton, Norway) to perform strain analysis in defined segments of the LV, and therefore, evaluate systolic and diastolic contractility. SrR were calculated at low and high levels of mPAP using speckle tracking analysis (EchoPAC PC Version 112; General Electric Co) from the short axis midpapillary view. The results are displayed as the differences with standard deviations (SD).

In addition, the integral of the transseptal pressure gradient (∫RVP‐LVP) (when RVP > LVP calculated according to Handoko et al. (2009) was used to evaluate the dimension of RV failure and the interventricular asynchrony.

### Statistics

Statistical analyses were performed using version 24 of SPSS Statistics software (IBM SPSS Statistics; IBM Corporation, Chicago, IL, USA).

A *P* < 0.05 was considered significant.

**Figure 1:**
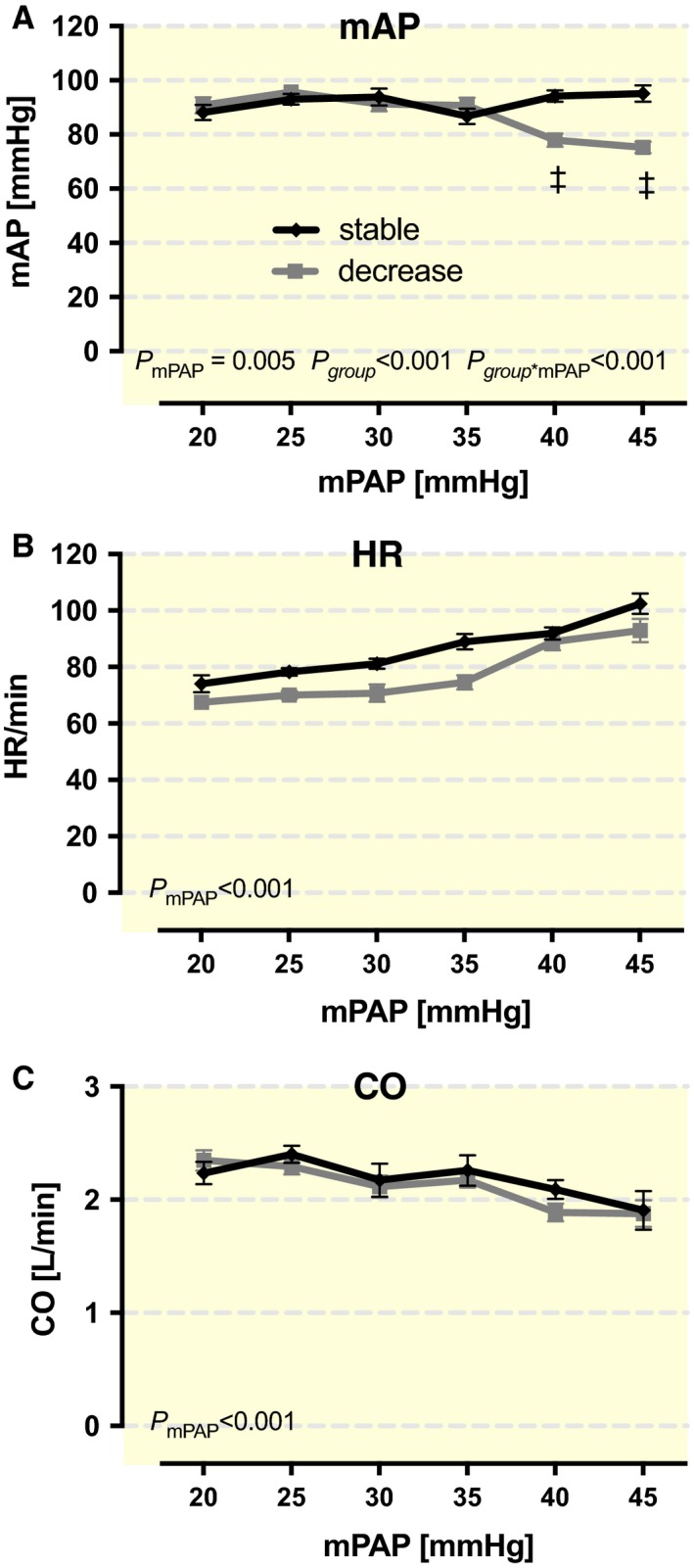
Influence of rising mean pulmonary artery pressure (mPAP) in both the stable group (SG) (*n* = 10) and the decrease group (DG) (*n* = 12) on (A) mean arterial pressure (mAP), (B) heart rate (HR) and (C) cardiac output (CO). *P*‐values within the figures were derived from ANOVA. Symbols mark significant differences between groups at the mPAP level (*<0.05; †<0.01; ‡<0.001).

The results are displayed in graphs after grouping mPAP values into groups of 5 mmHg starting with a group center of 20 mmHg (Fig. 1A). All figures were designed with GraphPad Prism 8.0 (GraphPad Software, Inc., San Diego, CA, USA).

Based on the mAP trend during the stepwise mPAP increase, animals were divided into two groups for further analysis. With an mAP decrease less than 5 mmHg, they were classified as “stable” group (SG) (*n* = 10). In all other cases, they were allocated to the decrease group (DG) (*n* = 12).

A generalized estimating equation for longitudinal data analysis was used as we conducted repeated measurements of the same objects at different points in time. Therefore, the animal number that uniquely identifies the individuals served as the subject variable, and the time of measurement served as the within‐subject variable. The binary grouped mAP data and the mPAP levels constituted the predictors. The effects of these independent variables on different parameters, including HR, CO, elastance, relaxation, ventricular volumes, transseptal pressure gradient, interventricular asynchronism, CBF and ST segment elevation, were described by Wald statistics and *P*‐values. Significant differences between the stable and decreased mAP groups were further analyzed using t‐tests for grouped parameters.

Delta SrRs (SrR at high level mPAP minus SrR at low level mPAP) of the different groups at the different cardiac segments were compared using repeated analyses of variance (ANOVA). Differences in the individual groups were further analyzed using post hoc tests.

## Results

Gradually increased mPAP loads initially induce similar mAP trends in all animals (Fig. 1A). However, mPAP values greater or equal to 37.5 mmHg cause two significantly different reactions in mAP (*P* < 0.001) (Fig. 1A).

Ten animals showed a steady, or even marginal, rise in mAP of 2.9 ± 3.0 mmHg at maximum mPAP conditions. They were subsequently allocated to the SG (*n* = 10). An mPAP‐dependent mAP reduction in up to 16.4 ± 9.9 mmHg was observed in the other 12 animals, which were allocated to the DG (*n* = 12).

In contrast, HR and CO changed concordantly in both groups. There was a significant increase in HR (Fig. 1B) and a significant decrease in CO (Fig. 1C) with rising mPAP (*P* < 0.001).

**Figure 2:**
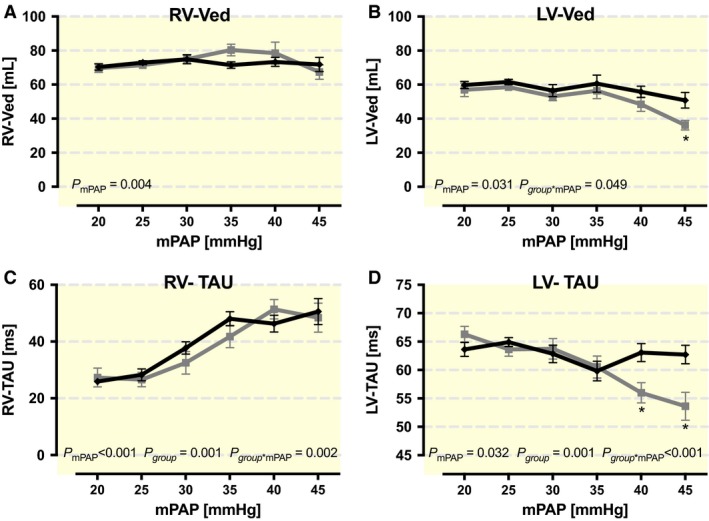
Changes in (A), (B) end‐diastolic volume (Ved) and (C), (D) time of isovolumetric relaxation (Tau) in the left (LV) as well as in the right ventricle (RV) due to acute pulmonary hypertension (rising mean arterial pressure (mAP)) in the two different groups. *P*‐values within the figures were derived from ANOVA. Symbols mark significant differences between groups at the mPAP level (*<0.05; †<0.01; ‡<0.001).

Pulmonary hypertension led to a reduced preload in the LV and a decreased end‐diastolic volume (Ved) (Fig. 2B). The loss of preload was stronger in the DG, and the difference was significant with mPAP values ≥42.5 mmHg (*P* = 0.031). The RV end‐diastolic volume (RV‐Ved) initially rose in both groups, and no significant differences in preload were observed (Fig. 2A).

Moreover, RV‐Tau (Greek letter *τ* (Tau) denotes the time constant for isovolumetric relaxation) increased with mPAP augmentation from 27.3 ± 9.9 ms (DG) and 25.9 ± 2.6 ms (SG) to 48.4 ± 13.6 ms (DG) and 50.6 ± 13.7 ms (SG), which are almost two times longer (Fig. 2C). There were no significant differences between the groups. In contrast, LV‐Tau showed a small decrease due to rising mPAP values (DG: 66.3 ± 4.1 ms to 53.6 ± 6.5 ms; SG: 63.6 ± 4.1 ms to 62.7 ± 4.9 ms) with significant differences between groups at mPAP levels greater than or equal to 37.5 mmHg (*P* = 0.001) (Fig. 2D). Consequently, the LV relaxation time with high mPAP levels was the shortest in the DG.

**Figure 3:**
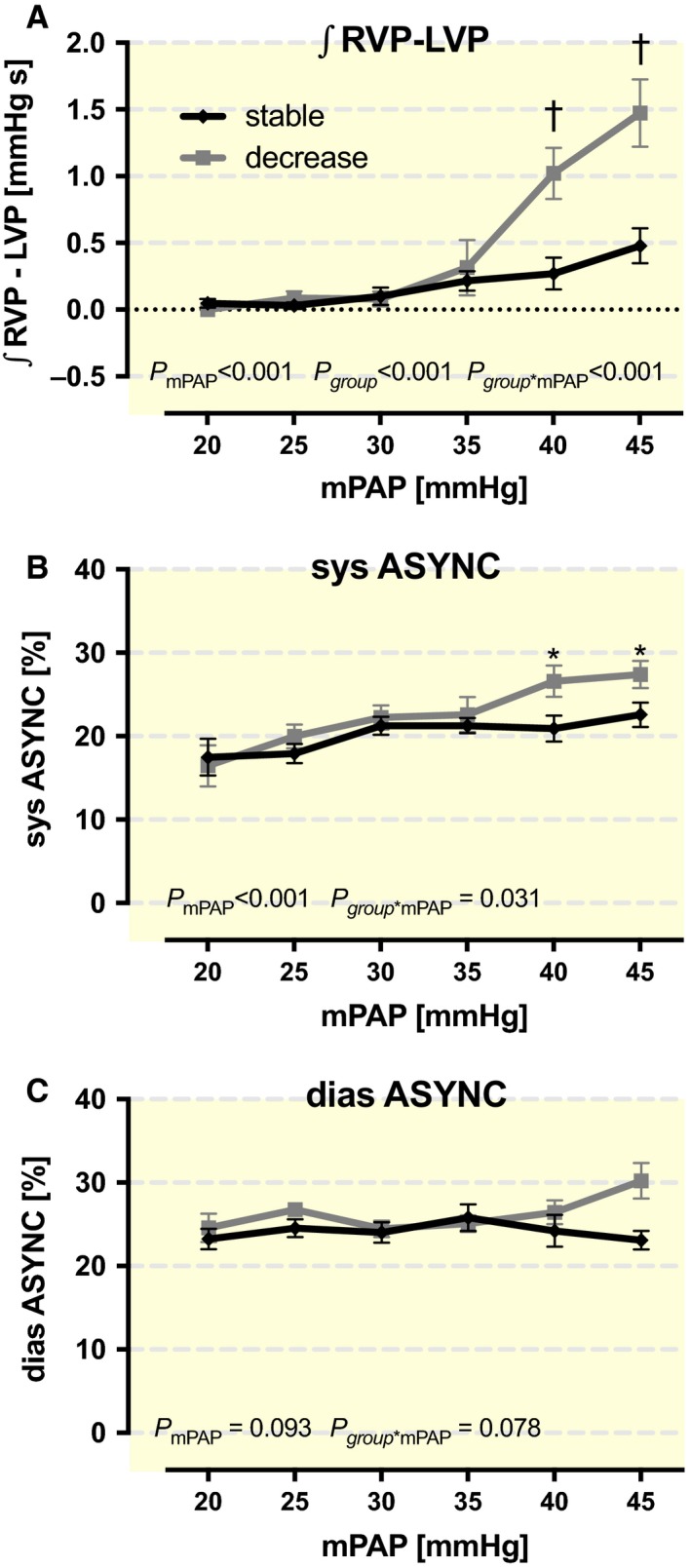
Interventricular synchronization with augmenting mean pulmonary artery pressure (mPAP) in the different groups. (A) The increasing integral of the difference between right and left ventricular pressure (∫RVP‐LVP) reflects a loss of synchronization. (B) Systolic and (C) diastolic asynchronism are pictured separately. *P*‐values within the figures were derived from ANOVA. Symbols mark significant differences between groups at the mPAP level (*<0.05; †<0.01; ‡<0.001).

Augmentation of the mPAP caused increased ∫_RVP‐LVP_ values (Fig. 3A). This change reflects the loss of synchronization based on progressive RV insufficiency and a larger septum bulging to the RV. For all mPAP stages greater than or equal to 37.5 mmHg, statistical analyses showed significant differences in ∫_RVP‐LVP_ between the observed groups (*P* < 0.001) (Fig. 3A). Furthermore distinction between the different contraction phases indicates that there was significance for the systolic asynchrony (*P* < 0.001) (Fig. 3B) but not for the diastolic asynchrony (*P* = 0.093) (Fig. 3C).

**Figure 4:**
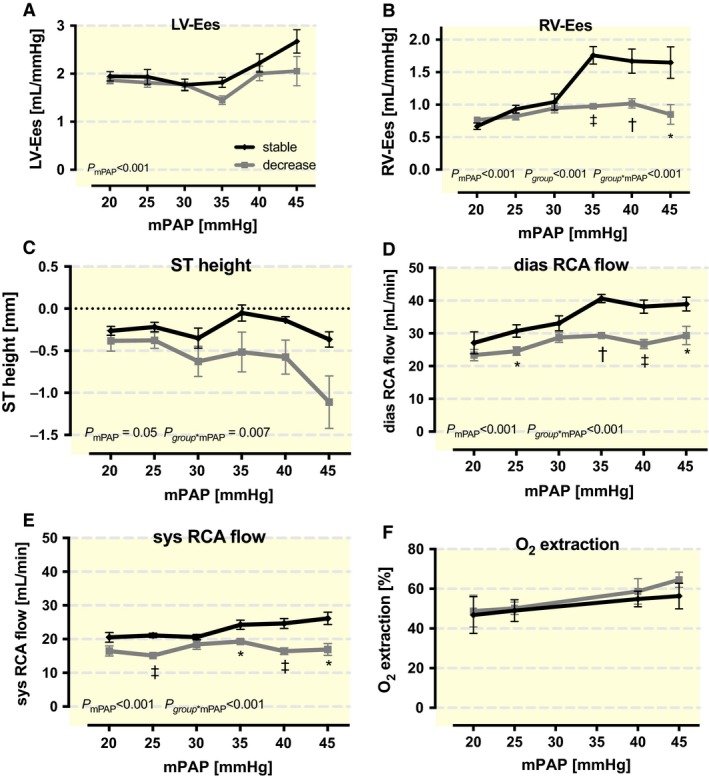
Different reactions to rising mean pulmonary artery pressures (mPAP) in the stable (SG) and the decrease group (DG) in (A) left (LV‐Ees) and (B) right ventricular end‐systolic elastance (RV‐Ees) as markers for contractility, (C) ST height, (D) diastolic (dias RCA flow) and (E) systolic right coronary artery flow (sys RCA flow) and (F) O_2_ extraction. *P*‐values within the figures were derived from ANOVA. Symbols mark significant differences between groups at the mPAP level (*<0.05; †<0.01; ‡<0.001).

With ascending mPAP values, the RCA flow in the SG increased both during systole (by 27%) (Fig. 4E) and diastole (by 44%) (Fig. 4D). Changes in the DG were considerably smaller (3% in systole and 26% in diastole) (Fig. 4D–E). Thus, the difference in the coronary flow increase between the groups was larger in diastole (44% vs. 26%) than in systole (27% vs. 3%). The absolute values of the two groups significantly differed at mPAP values of 25 mmHg and with values greater than or equal to 32.5 mmHg (*P* < 0.001) (Fig. 4D–E).

The missing CBF gain in the DG correlated with a significant, mPAP‐dependent increase in ST segment depression that was much greater than in the SG (p_group*mPAP _= 0.007) (Fig. 4C).

The myocardial oxygen consumption (MvO_2_) and the coronary oxygen extraction between the groups did not differ significantly. Consumption and extraction both slightly rose with the increase in mPAP (Fig. 4F).

Furthermore, the observed right CBF changes during PHT, which corresponds to the RV contractility that only increased in the SG. Differences between the groups were significant at mPAP values of 32.5 mmHg and above (*P* < 0.001) (Fig. 4B). As a preload‐independent measure of ventricular contractility, end‐systolic elastance (Ees) was used.

The LV contractility increased as well, but the differences between groups were not significant (Fig. 4A).

**Figure 5:**
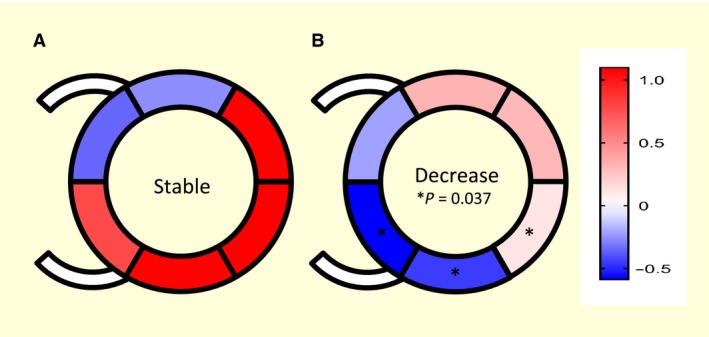
Analysis of echocardiographic left ventricular data of 17 animals: the change in the radial strain rate (SrR) [1/second] in a short axis in separate segments during rest (mean pulmonary artery pressure (mPAP) 20–25 mmHg) is illustrated and compared to pulmonary artery hypertension (mPAP 40–45 mmHg). Significant differences (*P < 0.05) between the two groups (A) Stable group; (B) Decrease group) were seen in the posterior septal wall as well as the inferior and posterior left ventricular free wall.

To evaluate the echocardiographic LV data of 17 animals (SG: *n* = 8; DG: *n* = 9), basal segmental radial strain rates (SrRs) (mPAP 20–25 mmHg) were subtracted from SrRs during PHT (mPAP 40–45 mmHg). The generated differences are illustrated in Figure 5. The DG showed a significantly smaller increase or even a decline in contractility in the mentioned areas compared to the SG (Fig. 5).

## Discussion

Infusion of the thromboxane A_2_ analogue U46619 induced PHT stepwise and led to adaptation that was classified into two different groups of animals: the SG (*n* = 10) with approximately constant mAP values and the DG (*n* = 12) with significantly reduced mAP values at higher mPAP levels (≥37.5 mmHg). In our comparison, the SG showed beside the stable mAP values, a larger increase in RCA flow, a gain of RV contractility, less pronounced ST depressions, a reduced loss of inter‐ventricular asynchrony and longer LV relaxation times. Overall adaptation was considerably better in the SG.

At the onset of significant group differences, SG and DG separate in RV contractility and RCA flow both in systole and in diastole (at mPAP ≥32.5 mmHg). Interestingly, mAP differences appear later (at mPAP ≥37.5 mmHg). Therefore, the mAP increase cannot be the decisive factor for the observed CBF gain in our experiments. The wall tension could also not have caused the observed CBF gain because elevated RV wall tensions during PHT would deteriorate instead of elevate CBF (Lowensohn et al. 1976; Gibbons Kroeker et al. 2006).

Because CBF has been described as an essential factor that limits or allows changes in myocardial contractility (Brooks et al. 1971; Klima et al. 1999; Zong et al. 2005), we presume that the CBF change determined the contractility alteration. In contrast, Belenkie et al. (1995) did not find a correlation between RCA flow and contractility in dogs by controlling RCA pressure, keeping in mind that the coronary pattern in dogs is very different from that in humans and pigs because dogs have more collaterals, and usually, the LCA is dominant and does supply 70–75% of the interventricular septum (Weaver et al. 1986). Furthermore, Belenkie et al. did not measure coronary oxygen extraction. The artificially controlled RCA pressure may have led to different extraction values than those in this study.

However, the extent to which RV autoregulation can maintain coronary blood pressure (CBP) has not yet been determined. A stable flow down to CBPs of 30 mmHg in pigs has been described (Guth et al. 1991). These results could have been incorrect due to baroreflex because both carotid arteries were ligated (Zong et al. 2005). Coronary perfusion pressure and flow in the RV are closely correlated with MvO_2_ (Yonekura et al. 1987; Zong et al. 2005). Therefore, to prevent unnecessary MvO_2_, the attempt to maintain coronary flow, despite pressure changes, using autoregulation appears to be appropriate. In contrast, findings in dogs suggest there is very limited RV coronary pressure‐flow autoregulation (Murray and Vatner 1981; Urabe et al. 1985; Yonekura et al. 1988; Bian et al. 1998; Zong et al. 2005). This result could be due to species‐specific differences.

Therefore, the question becomes what influenced the changes in RV coronary muscle tone in our experiments. Three crucial influencing components for CBF regulation have been described: local metabolites, *α*‐adrenoreceptors and ß‐adrenoreceptors (Gorman et al. 1985).

One decisive factor among the metabolites is NO. It has been demonstrated that NO causes vasodilatation and maintains basal RV‐CBF (Shen et al. 1985; Benyo et al. 1991; Sonntag et al. 1992; Duncker et al. 2000; Setty et al. 2002; Zong et al. 2002). In contrast, NO influence on the LV is quite small because it causes only slight redistribution of blood to the outer wall layers (Sonntag et al. 1992; Bernstein et al. 1996; Duncker et al. 2000). Interestingly, different RV strategies have been observed in animal experiments for handling variable causes of increased O_2_ demand (e.g., during PHT and exercise) (Zong et al. 2005). The RV responds to a greater O_2_ requirement during exercise initially using its great O_2_ extraction reserve, and only when venous pO_2_ falls below 20 mmHg does CBF increase in the left and right coronary artery (Tune et al. 1985; Hart et al. 2001; Zong et al. 2002). In contrast, PHT causes an early increase in CBF that provides up to 82% of the elevated oxygen consumption without first using the O_2_ extraction reserve (Zong et al. 2005). This result matches our findings because the O_2_ extraction with PHT was only slightly elevated to 65% in the DG and 56% in the SG. This group difference was not significant but could point to a better ability for increasing O_2_ extraction that could possibly improve the outcome by avoiding ischemia. Zong et al. (2005) postulated in their PHT trials that pulmonary balloon occlusion did not reduce mAP, and therefore, no sympathetic activation via baroreceptors occurred that would normally have blunted NO‐mediated vasodilatation during exercise. This possible mechanism would not explain our results. The changes in HR, as a parameter for sympathetic activation, do not inversely correlate with CBF changes in any group. Indeed, with rising mPAP, the DG showed a falling mAP (Fig. 1A), and only in this group did initially increased RCA flow decline as well (Fig. 4D–E). However, as explained above, the onset of the changes caused us to hypothesize that the lack of CBF gain during rising loads causes the falling mAP in the DG rather than the other way round.

As previously discussed, the increased sympathetic activity is *α*‐adrenoceptor‐mediated and works in opposite to the NO‐mediated vasodilatation. The importance of the sympathetic nervous system during PHT has already been demonstrated because a sympathetic blockade using thoracic epidural anesthesia in pigs seriously impaired the adaptation to PHT (Rex et al. 2007). Furthermore, the LV showed an initial, and probably catecholamine‐triggered, increase in contractility followed by a subsequent LV failure in reaction to PHT (Chua et al. 2013). Sympathetic activity is important in the RV because the RV at rest only extracts 43–50% of O_2_ of the coronary vessels (whereas 60–75% is extracted in the LV) (von Restorff et al. 1977; Kusachi et al. 1982; Tune et al. 1985; Takeda et al. 1987; Saito et al. 1989). Lower O_2_ requirements and less systolic internal wall pressure with a consequent increase in systolic CBF are assumed to be reasons for the low right coronary extraction rate compared to that of the left side (Lowensohn et al. 1976; Hess and Bache 1979). Through vasoconstriction, sympathetic activation ensures that during exercise, the substantial O_2_ extraction reserve is being used, and thus, RV function is preserved during reduced perfusion pressure (Zong et al. 2005). The described extraction values match our own results. Similarly, in our animals, a sympathetic activation can be assumed due to the rising HR. Appropriately, the O_2_ extraction rose with increasing mPAP and MvO_2_ in both groups (Fig. 4F).

Sympathetic activation during exercise also results in ß‐adrenoreceptor‐mediated vasodilatation (Gorman et al. 1985; Tune et al. 1985). Thus, an adequate oxygen supply with rising oxygen demand is ensured.

Additional controllers, including adenosine, K_ATP_ channels, prostaglandins, endothelium‐derived hyperpolarizing factor and endothelin, have been discussed (Tune et al. 1985).

Coronary blood flow regulation is a complex mechanism that is, especially in specific situations like PHT, not yet completely understood. Furthermore research on this subject is needed.

Returning to the chronological order of changes during PHT, one sees that the indices of interventricular asynchrony differ between the groups in LV diastolic function and occur only with higher PHT (mPAP ≥37.5 mmHg), which is similar to the differences in mAP. Finally (mPAP ≥42.5 mmHg) LV preload exhibited significantly different values in the groups, which indicates a distinct right heart failure with venous congestion in the DG.

It is characteristic for the RV that HR, afterload and contractility dictate the O_2_ demand (Zong et al. 2005).

Because both ventricles share the septal wall, they affect each other directly. Therefore, when assessing RV function, it is necessary to consider ventricular interdependence as well. Therefore, mainly ventricular asynchrony leads to great changes in physiological characteristics.

There is an increase in RV‐Tau during PHT, whereas LV‐Tau lessens simultaneously (Fig. 2C–D), that illustrates the loss of interventricular synchrony. Therefore, PHT clearly elongates isovolumic relaxation time in the RV and shortens it in the LV. As previously described, this process allows the septum to bulge to the left during early diastole. The bulging septum as well as a missing ventricular inflow into the left ventricle due to the PHT could have caused the observed reduced LV‐end‐diastolic volume with increased mPAP.

Appropriately, falling CO, that is known to be sign of RV decompensation, occurs with increasing mPAP values (Fig. 1C) (Forrest 2009).

In addition, calculating ∫_RVP‐LVP_ provides reliable information concerning RV function because it demonstrates duration and quantity of the reversed transseptal pressure gradient. It is important to note that the findings depend on the RV volume state (Handoko et al. 2009). As expected, the results show that right heart failure at high pulmonary pressure levels was more distinctive in the DG than in the stable one.

How far the loss of ventricular synchronism provokes right heart failure or if RV failure in fact causes the delay has not been entirely clarified.

Furthermore reliable examination of interventricular interaction can be done regarding echocardiography and septal strain analysis.

Pulmonary hypertension has been demonstrated to provoke only septal a reduced radial and circumferential strain (Chua et al. 2013). As a potential cause for the regional differences, septum bulging with a consequent loss of the helical myocardial structure and the inability for the usual torsion to occur have been discussed (Chua et al. 2013). This theory could not be confirmed using our data because only the DG showed a reduced septal strain rate (Fig. 5).

Analysis of the whole LV echocardiographic data particularly underlines the significant differences in contractility changes between the groups in the posterior septal wall and the inferior posterior LV free wall (Fig. 5). The proximity of these two segments led to the assumption that differences in blood flow could be the cause for the discrepancies. Although coronary supply territories in swine are described as very uniform, some interindividual variants have been described (Weaver et al. 1986; Guth et al. 1991). One example delineates whether a small part of the LV posterior wall is supplied with blood via the left or right coronary artery (Guth et al. 1991). A closely related example is the posterior septal wall that is normally supplied via the RCA (Guth et al. 1991). Variations in coronary blood supply in these areas and, therefore, a different pattern of coronary border zones could cause the observed differences in contractility changes during increased stress from PHT. At the same time, the occurrence and formation of collaterals that accompanied their flow direction could possibly play an important role as well because a well‐organized collateral system can ensure the perfusion of critical segments even during increasing demands (Gatzov et al. 2003). Regarding the transferability of these findings to humans, many studies showed greater similarities than differences between the species (Weaver et al. 1986; Rodrigues et al. 2005). For example, there were comparatively few collaterals (Weaver et al. 1986), and roughly the same courses were followed (Rodrigues et al. 2005). However, there are some differences, such as a predominance of the RCA in humans in contrast to a balanced distribution in swine (Weaver et al. 1986; Rodrigues et al. 2005).

### Limitations

We focused on the examination of acute PHT so that our statements are limited to this specific event because the time course plays an important role in the pathogenesis of the disease. Moreover, studying one animal species can lead to problems with transferability in cases of existing interspecies variability. Furthermore, the experimental setup itself could have influenced the results. For example, general anesthesia influences the cardiovascular system in different ways. It has been demonstrated that it can possibly modify coronary muscle tone (Vatner and Braunwald 1975), limit coronary autoregulation range (Bian et al. 1998), reduce CO and induce hypotension (Berthoud and Reilly 1992). Similarly, the opened chest and pericardium are deviations of physiological conditions that facilitate cardiac filling and reduce the influence of LV septal bowing. Besides the thromboxane A_2_ analogue U46619 infusion itself might have had a small systemic effect, but as the two groups did not differ significantly in the dosage of U46619 needed and the CO, the drug could not explain the observed major differences in mAP. Moreover an influence of U46619 on the inotropy of the heart cannot be ruled out completely, but given the divergent effects on the different wall regions (Fig. 5) it is very unlikely.

## Conclusion

In summary, our data suggest that the increasing CBF is essential for patient adaptation to acute PHT. The decisive factor for adaptation to rising pulmonary pressure could be the differences in coronary supply territories.

Indeed, our findings may suggest that further research, especially with regard to reasons and possible signs for early detection of CBF rise, is needed.

## Conflicts of interest

The authors declare that they have no conflicts of interest regarding the publication of this paper.

## References

[phy213605-bib-0001] Ama, R. , H. A. Leather , P. Segers , E. Vandermeersch , and P. F. Wouters . 2006 Acute pulmonary hypertension causes depression of left ventricular contractility and relaxation. Eur. J. Anaesthesiol. 23:824–831. 16953943 10.1017/S0265021506000317

[phy213605-bib-0002] Baan, J. , E. T. van der Velde , H. G. de Bruin , G. J. Smeenk , J. Koops , A. D. van Dijk , et al. 1984 Continuous measurement of left ventricular volume in animals and humans by conductance catheter. Circulation 70:812–823. 6386218 10.1161/01.cir.70.5.812

[phy213605-bib-0003] Belenkie, I. , S. G. Horne , R. Dani , E. R. Smith , and J. V. Tyberg . 1995 Effects of aortic constriction during experimental acute right ventricular pressure loading. Further insights into diastolic and systolic ventricular interaction. Circulation 92:546–554. 7634469 10.1161/01.cir.92.3.546

[phy213605-bib-0004] Benyo, Z. , G. Kiss , C. Szabo , C. Csaki , and A. G. Kovach . 1991 Importance of basal nitric oxide synthesis in regulation of myocardial blood flow. Cardiovasc. Res. 25:700–703. 1913760 10.1093/cvr/25.8.700

[phy213605-bib-0005] Bernstein, R. D. , F. Y. Ochoa , X. Xu , P. Forfia , W. Shen , C. I. Thompson , et al. 1996 Function and production of nitric oxide in the coronary circulation of the conscious dog during exercise. Circ. Res. 79:840–848. 8831509 10.1161/01.res.79.4.840

[phy213605-bib-0006] Berthoud, M. C. , and C. S. Reilly . 1992 Adverse effects of general anaesthetics. Drug Saf. 7:434–459. 1418699 10.2165/00002018-199207060-00005

[phy213605-bib-0007] Bian, X. , A. G. Jr Williams , P. A. Gwirtz , and H. F. Downey . 1998 Right coronary autoregulation in conscious, chronically instrumented dogs. Am. J. Physiol. 275:H169–H175. 9688910 10.1152/ajpheart.1998.275.1.H169

[phy213605-bib-0008] Bleeker, G. B. , P. Steendijk , E. R. Holman , C. M. Yu , O. A. Breithardt , T. A. Kaandorp , et al. 2006 Acquired right ventricular dysfunction. Heart 92(Suppl 1):i14–i18. 16543596 10.1136/hrt.2005.081547 PMC1860732

[phy213605-bib-0009] Bristow, M. R. , L. S. Zisman , B. D. Lowes , W. T. Abraham , D. B. Badesch , B. M. Groves , et al. 1998 The pressure‐overloaded right ventricle in pulmonary hypertension. Chest 114:101S–106S. 9676654 10.1378/chest.114.1_supplement.101s

[phy213605-bib-0010] Brooks, H. , E. S. Kirk , P. S. Vokonas , C. W. Urschel , and E. H. Sonnenblick . 1971 Performance of the right ventricle under stress: relation to right coronary flow. Exp Biol Med 50:176–183. 10.1172/JCI106712 PMC292152 5116207

[phy213605-bib-0011] Chua, J. H. , W. Zhou , J. K. Ho , N. A. Patel , G. B. Mackensen , and A. Mahajan . 2013 Acute right ventricular pressure overload compromises left ventricular function by altering septal strain and rotation. J. Appl. Physiol. 115:186–193. 23661621 10.1152/japplphysiol.01208.2012 PMC3727005

[phy213605-bib-0012] Crottogini, A. J. , B. D. Guth , J. G. Barra , P. Willshaw , E. C. Lascano , and R. H. Pichel . 1991 Interventricular coronary steal induced by stenosis of left anterior descending coronary artery in exercising pigs. Circulation 83:1361–1370. 2013153 10.1161/01.cir.83.4.1361

[phy213605-bib-0013] Danton, M. H. , J. G. Byrne , M. Hsin , R. Laurence , L. H. Cohn , and L. Aklog . 2002 Right ventricular volume measurement using the conductance catheter method: validation in excised porcine hearts. ASAIO J. 48:514–519. 12296572 10.1097/00002480-200209000-00013

[phy213605-bib-0014] Duncker, D. J. , R. Stubenitsky , P. A. Tonino , and P. D. Verdouw . 2000 Nitric oxide contributes to the regulation of vasomotor tone but does not modulate O(2)‐consumption in exercising swine. Cardiovasc. Res. 47:738–748. 10974222 10.1016/s0008-6363(00)00143-7

[phy213605-bib-0015] Fischer, L. G. , H. Van Aken , and H. Burkle . 2003 Management of pulmonary hypertension: physiological and pharmacological considerations for anesthesiologists. Anesth. Analg. 96:1603–1616. 12760982 10.1213/01.ANE.0000062523.67426.0B

[phy213605-bib-0016] Forrest, P. 2009 Anaesthesia and right ventricular failure. Anaesth. Intensive Care 37:370–385. 19499856 10.1177/0310057X0903700314

[phy213605-bib-0017] Gatzov, P. , A. Manginas , V. Voudris , G. Pavlides , G. D. Genchev , and D. V. Cokkinos . 2003 Blood flow velocity in donor coronary artery depends on the degree and pattern of collateral vessel development: a study using thrombolysis in myocardial infarction frame count method. Catheter. Cardiovasc. Interv. 60:462–468. 14624422 10.1002/ccd.10694

[phy213605-bib-0018] Gibbons Kroeker, C. A. , S. Adeeb , N. G. Shrive , and J. V. Tyberg . 2006 Compression induced by RV pressure overload decreases regional coronary blood flow in anesthetized dogs. Am. J. Physiol. Heart Circ. Physiol. 290:H2432–H2438. 16428352 10.1152/ajpheart.01140.2005

[phy213605-bib-0019] Goldstein, J. A. 2002 Pathophysiology and management of right heart ischemia. J. Am. Coll. Cardiol. 40:841–853. 12225706 10.1016/s0735-1097(02)02048-x

[phy213605-bib-0020] Gorman, M. W. , J. D. Tune , K. N. Richmond , and E. O. Feigl . 1985 Feedforward sympathetic coronary vasodilation in exercising dogs. J. Appl. Physiol. 89(1892–902):2000. 10.1152/jappl.2000.89.5.1892 11053341

[phy213605-bib-0021] Guth, B. D. , R. Schulz , and G. Heusch . 1991 Pressure‐flow characteristics in the right and left ventricular perfusion territories of the right coronary artery in swine. Pflugers Arch. 419:622–628. 1788057 10.1007/BF00370305

[phy213605-bib-0022] Handoko, M. L. , R. R. Lamberts , E. M. Redout , F. S. de Man , C. Boer , W. S. Simonides , et al. 2009 Right ventricular pacing improves right heart function in experimental pulmonary arterial hypertension: a study in the isolated heart. *AJP: Heart and Circulatory* . Physiology 297:H1752–H1759. 10.1152/ajpheart.00555.2009 19734361

[phy213605-bib-0023] Hardziyenka, M. , M. E. Campian , B. J. Bouma , A. C. Linnenbank , H. A. Bruin‐Bon , J. J. Kloek , et al. 2009 Right‐to‐left ventricular diastolic delay in chronic thromboembolic pulmonary hypertension is associated with activation delay and action potential prolongation in right ventricle. Circ. Arrhythm. Electrophysiol., 2:555–561. 19843924 10.1161/CIRCEP.109.856021

[phy213605-bib-0024] Hart, B. J. , X. Bian , P. A. Gwirtz , S. Setty , and H. F. Downey . 2001 Right ventricular oxygen supply/demand balance in exercising dogs. Am. J. Physiol. Heart Circ. Physiol. 281:H823–H830. 11454587 10.1152/ajpheart.2001.281.2.H823

[phy213605-bib-0025] Hess, D. S. , and R. J. Bache . 1979 Transmural right ventricular myocardial blood flow during systole in the awake dog. Circ. Res. 45:88–94. 445700 10.1161/01.res.45.1.88

[phy213605-bib-0026] Institute of Laboratory Animal Resources CoLS, National Research Council 1996 Guide for the Care and Use of Laboratory Animals. 7th ed National Academies Press, Washington, DC.

[phy213605-bib-0027] Kaw, R. , V. Pasupuleti , A. Deshpande , T. Hamieh , E. Walker , and O. A. Minai . 2011 Pulmonary hypertension: an important predictor of outcomes in patients undergoing non‐cardiac surgery. Respir. Med. 105:619–624. 21195595 10.1016/j.rmed.2010.12.006

[phy213605-bib-0028] Klima, U. P. , J. L. Guerrero , and G. J. Vlahakes . 1999 Myocardial perfusion and right ventricular function. Ann. Thorac. Cardiovasc. Surg. 5:74–80. 10332109

[phy213605-bib-0029] Kusachi, S. , O. Nishiyama , K. Yasuhara , D. Saito , S. Haraoka , and H. Nagashima . 1982 Right and left ventricular oxygen metabolism in open‐chest dogs. Am. J. Physiol. 243:H761–H766. 7137369 10.1152/ajpheart.1982.243.5.H761

[phy213605-bib-0030] Lai, H. C. , H. C. Lai , K. Y. Wang , W. L. Lee , C. T. Ting , and T. J. Liu . 2007 Severe pulmonary hypertension complicates postoperative outcome of non‐cardiac surgery. Br. J. Anaesth. 99:184–190. 17576968 10.1093/bja/aem126

[phy213605-bib-0031] Lowensohn, H. S. , E. M. Khouri , D. E. Gregg , R. L. Pyle , and R. E. Patterson . 1976 Phasic right coronary artery blood flow in conscious dogs with normal and elevated right ventricular pressures. Circ. Res. 39:760–766. 1000768 10.1161/01.res.39.6.760

[phy213605-bib-0032] Marcus, J. T. , C. T.‐J. Gan , J. J. M. Zwanenburg , A. Boonstra , C. P. Allaart , M. J. W. Götte , et al. 2008 Interventricular Mechanical Asynchrony in Pulmonary Arterial Hypertension. J. Am. Coll. Cardiol. 51:750–757. 18279740 10.1016/j.jacc.2007.10.041

[phy213605-bib-0033] Mebazaa, A. , P. Karpati , E. Renaud , and L. Algotsson . 2004 Acute right ventricular failure–from pathophysiology to new treatments. Intensive Care Med. 30:185–196. 14618229 10.1007/s00134-003-2025-3

[phy213605-bib-0034] Murray, P. A. , and S. F. Vatner . 1981 Carotid sinus baroreceptor control of right coronary circulation in normal, hypertrophied, and failing right ventricles of conscious dogs. Circ. Res. 49:1339–1349. 6118210 10.1161/01.res.49.6.1339

[phy213605-bib-0035] Opitz, C. , S. Rosenkranz , H. A. Ghofrani , E. Grunig , H. Klose , H. Olschewski , et al. 2016 ESC guidelines 2015 pulmonary hypertension: diagnosis and treatment. Dtsch. Med. Wochenschr. 141:1764–1769. 27903027 10.1055/s-0042-117784

[phy213605-bib-0036] Pirat, B. , M. L. McCulloch , and W. A. Zoghbi . 2006 Evaluation of global and regional right ventricular systolic function in patients with pulmonary hypertension using a novel speckle tracking method. Am. J. Cardiol. 98:699–704. 16923465 10.1016/j.amjcard.2006.03.056

[phy213605-bib-0037] Reich, D. L. , C. A. Bodian , M. Krol , M. Kuroda , T. Osinski , and D. M. Thys . 1999 Intraoperative hemodynamic predictors of mortality, stroke, and myocardial infarction after coronary artery bypass surgery. Anesth. Analg. 89:814–822. 10512249 10.1097/00000539-199910000-00002

[phy213605-bib-0038] von Restorff, W. , J. Holtz , and E. Bassenge . 1977 Exercise induced augmentation of myocardial oxygen extraction in spite of normal coronary dilatory capacity in dogs. Pflugers Arch. 372:181–185. 564040 10.1007/BF00585334

[phy213605-bib-0039] Rex, S. , C. Missant , P. Segers , and P. F. Wouters . 2007 Thoracic epidural anesthesia impairs the hemodynamic response to acute pulmonary hypertension by deteriorating right ventricular‐pulmonary arterial coupling. Crit. Care Med. 35:222–229. 17095942 10.1097/01.CCM.0000250357.35250.A2

[phy213605-bib-0040] Rodrigues, M. S. A. C. , A. P. Aguas , and N. R. Grande . 2005 The coronary circulation of the pig heart: comparison with the human heart. Eur. J. Anat. 9:67–87.

[phy213605-bib-0041] Roehl, A. B. , P. Steendijk , R. Rossaint , C. Bleilevens , A. Goetzenich , and M. Hein . 2012 Xenon is not superior to isoflurane on cardiovascular function during experimental acute pulmonary hypertension. Acta Anaesthesiol. Scand. 56:449–458. 22260254 10.1111/j.1399-6576.2011.02624.x

[phy213605-bib-0042] Saito, D. , N. Yamada , S. Kusachi , H. Tani , A. Shimizu , K. Hina , et al. 1989 Coronary flow reserve and oxygen metabolism of the right ventricle. Jpn. Circ. J. 53:1310–1316. 2533279 10.1253/jcj.53.1310

[phy213605-bib-0043] Setty, S. , J. D. Tune , and H. F. Downey . 2002 Nitric oxide modulates right ventricular flow and oxygen consumption during norepinephrine infusion. Am. J. Physiol. Heart Circ. Physiol. 282:H696–H703. 11788420 10.1152/ajpheart.00398.2001

[phy213605-bib-0044] Shen, W. , M. Lundborg , J. Wang , J. M. Stewart , X. Xu , M. Ochoa , et al. 1985 Role of EDRF in the regulation of regional blood flow and vascular resistance at rest and during exercise in conscious dogs. J. Appl. Physiol. 77(165–72):1994. 10.1152/jappl.1994.77.1.165 7525527

[phy213605-bib-0045] Smulders, Y. M. 2000 Pathophysiology and treatment of haemodynamic instability in acute pulmonary embolism: the pivotal role of pulmonary vasoconstriction. Cardiovasc. Res. 48:23–33. 11033105 10.1016/s0008-6363(00)00168-1

[phy213605-bib-0046] Sonntag, M. , A. Deussen , and J. Schrader . 1992 Role of nitric oxide in local blood flow control in the anaesthetized dog. Pflugers Arch. 420:194–199. 1620578 10.1007/BF00374990

[phy213605-bib-0047] Steendijk, P. , and J. Baan . 2000 Comparison of intravenous and pulmonary artery injections of hypertonic saline for the assessment of conductance catheter parallel conductance. Cardiovasc. Res. 46:82–89. 10727656 10.1016/s0008-6363(00)00012-2

[phy213605-bib-0048] Takeda, K. , S. Haraoka , and H. Nagashima . 1987 Myocardial oxygen metabolism of the right ventricle with volume loading and hypoperfusion. Jpn. Circ. J. 51:563–572. 3626015 10.1253/jcj.51.563

[phy213605-bib-0049] Tune, J. D. , M. W. Gorman , and E. O. Feigl . 1985 Matching coronary blood flow to myocardial oxygen consumption. J. Appl. Physiol. 97(404–15):2004. 10.1152/japplphysiol.01345.2003 15220323

[phy213605-bib-0050] Urabe, Y. , H. Tomoike , K. Ohzono , S. Koyanagi , and M. Nakamura . 1985 Role of afterload in determining regional right ventricular performance during coronary underperfusion in dogs. Circ. Res. 57:96–104. 4006106 10.1161/01.res.57.1.96

[phy213605-bib-0051] Vatner, S. F. , and E. Braunwald . 1975 Cardiovascular control mechanisms in the conscious state. N. Engl. J. Med. 293:970–976. 1101063 10.1056/NEJM197511062931906

[phy213605-bib-0052] Vonk‐Noordegraaf, A. , J. T. Marcus , C. T. Gan , A. Boonstra , and P. E. Postmus . 2005 Interventricular mechanical asynchrony due to right ventricular pressure overload in pulmonary hypertension plays an important role in impaired left ventricular filling. Chest 128:628S–630S. 16373882 10.1378/chest.128.6_suppl.628S

[phy213605-bib-0053] Vonk‐Noordegraaf, A. , F. Haddad , K. M. Chin , P. R. Forfia , S. M. Kawut , J. Lumens , et al. 2013 Right heart adaptation to pulmonary arterial hypertension: physiology and pathobiology. J. Am. Coll. Cardiol. 62:D22–D33. 24355638 10.1016/j.jacc.2013.10.027

[phy213605-bib-0054] de Vroomen, M. , R. H. Cardozo , P. Steendijk , F. van Bel , and J. Baan . 2000 Improved contractile performance of right ventricle in response to increased RV afterload in newborn lamb. Am. J. Physiol. Heart Circ. Physiol. 278:H100–H105. 10644589 10.1152/ajpheart.2000.278.1.H100

[phy213605-bib-0055] Weaver, M. E. , G. A. Pantely , J. D. Bristow , and H. D. Ladley . 1986 A quantitative study of the anatomy and distribution of coronary arteries in swine in comparison with other animals and man. Cardiovasc. Res. 20:907–917. 3802126 10.1093/cvr/20.12.907

[phy213605-bib-0056] Yonekura, S. , N. Watanabe , J. L. Caffrey , J. F. Gaugl , and H. F. Downey . 1987 Mechanism of attenuated pressure‐flow autoregulation in right coronary circulation of dogs. Circ. Res. 60:133–141. 3568284 10.1161/01.res.60.1.133

[phy213605-bib-0057] Yonekura, S. , N. Watanabe , and H. F. Downey . 1988 Transmural variation in autoregulation of right ventricular blood flow. Circ. Res. 62:776–781. 3349576 10.1161/01.res.62.4.776

[phy213605-bib-0058] Zong, P. , J. D. Tune , S. Setty , and H. F. Downey . 2002 Endogenous nitric oxide regulates right coronary blood flow during acute pulmonary hypertension in conscious dogs. Basic Res. Cardiol. 97:392–398. 12200639 10.1007/s003950200048

[phy213605-bib-0059] Zong, P. , J. D. Tune , and H. F. Downey . 2005 Mechanisms of oxygen demand/supply balance in the right ventricle. Exp. Biol. Med. 230:507–519. 10.1177/153537020523000801 16118400

[phy213605-bib-0060] Zwissler, B. 2000 Acute right heart failure. Etiology–pathophysiology–diagnosis–therapy. Anaesthesist 49:788–808. 11076268 10.1007/s001010070052

